# The Health of London

**Published:** 1882-09

**Authors:** 


### THE HEALTH OF LONDON.

The death-rate of London during the two summer months reached the extraordinarily low figure of 13.45 per 1,000. That the city is less healthy in winter we might infer from the statement that the weight of the soot hanging over London on a winter's day is fifty tons, while that of the carbon dioxide is two hundred and fifty tons.

